# Endobronchial mucormycosis diagnosed by fiberoptic bronchoscopy

**DOI:** 10.1016/j.idcr.2023.e01781

**Published:** 2023-05-12

**Authors:** Hebatallah Hany Assal, Sabah Ahmed Hussein, Ahmed Mostafa, Dalia Abd El-Kareem, Mostafa Alfishawy, Maged Salah, Habiballah Galal Mohammed

**Affiliations:** a Department of Chest Medicine, Faculty of Medicine, Cairo University, Al Kasr Al Aini, Old Cairo, Cairo, Cairo Governorate 4240310, Egypt; b Cardiothoracic Surgery Department, Faculty of Medicine, Ain Shams University, Ramsis Street Square, El Weili, Cairo, Egypt; c Department of Pathology, Faculty of Medicine, Cairo University, Al Kasr Al Aini, Old Cairo, Cairo, Cairo Governorate 4240310, Egypt; d Infectious Diseases Consultants and Academic Researchers of Egypt (IDCARE), Cairo, Egypt; e Department of Anaethesia, Faculty of Medicine, Cairo University, Al Kasr Al Aini, Old Cairo, Cairo, Cairo Governorate 4240310, Egypt; f Radiodiagnosis Department, Misr International Hospital, 12 Ismail Abou El-Fotouh, Ad Doqi A, Dokki, Cairo, Giza Governorate 3753421, Egypt

**Keywords:** Endobronchial, Mucormycosis, Cryoprobe

## Abstract

**Introduction:**

Endobronchial mucormycosis is very rare with only few cases reported in the literature. Here, we report a rare presentation of pulmonary mucormycosis in a diabetic patient who presented with left lung collapse. Bronchoscopy revealed an endobronchial growth, mimicking a tumor, causing complete occlusion of the left main bronchus. Histopathology confirmed the diagnosis of invasive mucormycosis.

**Case presentation:**

Male patient 35 years old with accidental discovered Diabetes Mellitus, complained of hoarseness of voice and dry irritating cough that didn’t respond to antitussives and nonspecific treatment. CT chest was done and revealed left total lung collapse. Fiberoptic bronchoscopy was done and revealed total occlusion of the left main bronchus with whitish fungating glistening tissue from which biopsies were obtained. Histopathological examination was consistent with mucormycosis. A trial of medical treatment failed after which the patient was referred for surgical resection.

**Conclusion:**

Successful treatment of mucormycosis requires early diagnosis; prompt administration of antifungal therapy, and surgical intervention when applicable. Aggressive surgical intervention to remove necrotic tissue is generally accepted as the therapeutic mainstay for endobronchial obstructing mucormycosis

## Introduction

Pulmonary mucormycosis is a serious opportunistic fungal infection that occurs most commonly in immunocompromised but also reported in an immunocompetent host. Prolonged immunosuppression, poorly controlled diabetes mellitus, and hematological malignancies are considered the major risk factors [1]. Pulmonary lesions mostly tend to present radiologically as consolidation or cavitation and the patients usually present with nonspecific symptoms like cough, dyspnea, chest pain, and fever [2]. Endobronchial mucormycosis is very rare with only few cases reported in the literature [3], [4]. Bronchoscopic evaluation and histopathological examination of the lesions are necessary to differentiate it from a neoplasm. Here, we report a rare presentation of pulmonary mucormycosis in a diabetic patient who presented with left lung collapse. Bronchoscopy revealed an endobronchial growth, mimicking a tumor, causing complete occlusion of the left main bronchus. Histopathology confirmed the diagnosis of invasive mucormycosis.

## Case presentation

**Fig. 1:**
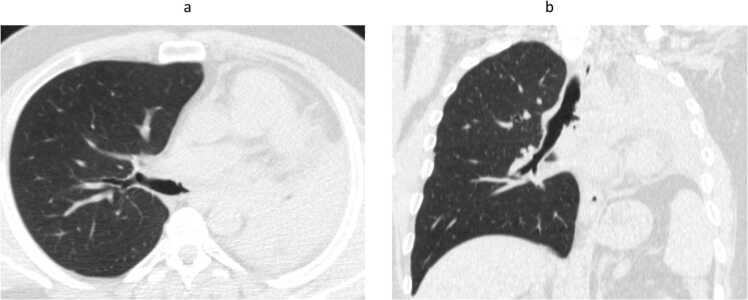
Axial and coronal CT images in lung window at the level of the carina. Axial (a)and coronal (b) CT images in lung window at the level of the carina showing total obliteration of the left main bronchus by an iso dense lesion, with subsequent total collapse of the left lung and minimal left pleural effusion.

**Fig. 2:**
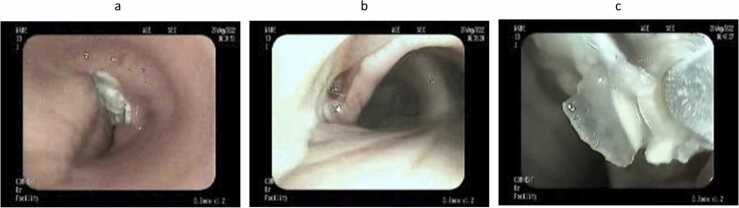
Bronchoscopic images of the lesion. a-whitish tumor tissue is seen protruding from the left main bronchus and creeping on the carina, b-carina is eroded by the tumor, c- a cryoprobe is attached to the tumor during taking the biopsies.

**Fig. 3:**
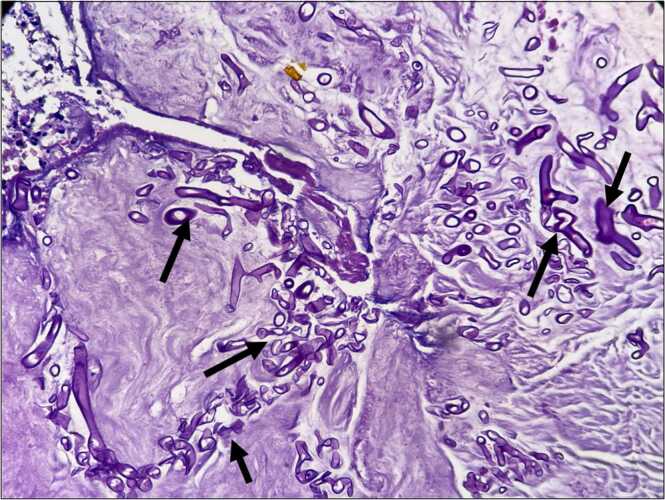
Histopathology of the tissue. Photomicrograph showing mucoid and necrotic material infiltrated by broad ribbon-like pauciseptate hyphae, irregularly branching at 90°. (H&E stain, original magnification x 100).

Male patient 35 years old with accidental discovered Diabetes Mellitus, admitted in the hospital with diabetic Ketoacidosis. On admission, the patient had no chest complaint. A pre-admission CT chest was done as this was the hospital's policy during the COVID pandemic and revealed to be free. The patient was discharged on insulin and followed up in the endocrinology clinic. After 1 month he started to complain of hoarseness of voice and dry irritating cough that didn’t respond to antitussives and nonspecific treatment. CT chest was done and revealed left total lung collapse (Fig. 1). Fiberoptic bronchoscopy was done and revealed total occlusion of the left main bronchus with whitish fungating glistening tissue. Endobronchial biopsies with cryoprobe were performed (Fig. 2). Multiple attempts of tumor extraction with the cryoprobe was done. However, the attempts were unsuccessful as the mass was very elastic, and firmly attached to the underlying lung tissue. Biopsies were sent for fungal tissue culture as well as histopathology. Fungal tissue culture revealed to be negative. Histopathological examination of the biopsies revealed hyalinized fibrous tissue, granulation tissue, necrotic material and debris infiltrated by large number of broad ribbon like puaciseptate hyphae, irregularly branching at 90 degrees which was consistent with mucormycosis (Fig. 3). The patient received liposomal amphotericin B 5 mg/Kg for 2 weeks. Repeated bronchoscopies as well as Chest imaging didn’t show signs of improvement. A trial of mechanical debulking of the tissue was done through rigid bronchoscope but was unsuccessful. Finally, the patient was referred to cardiothoracic surgery.

After intubation, venovenous ECMO (Cardiohelp, Maquet) was connected to the patient. Right femoral vein and right internal jugular vein were canulated with 27 French and 19 French canula respectively and gas flow was adjusted to 3–4 liters/minute to achieve oxygen saturation of 88–92%.

A clamshell incision was done (bilateral anterolateral thoracotomies plus transverse sternotomy), dissection of right and laft main bronchus was done, exploration revealed destruction of the whole length of the left main bronchus, so a decision of left carinal sleeve pneumonectomy was taken.

The distal trachea was cut, left pneumonectomy was done and the right main bronchus was anastomosed to the distal trachea. Extensive adhesions were encountered especially between the left main bronchus and the left pulmonary artery making it an extremely challenging lengthy operative procedure that lasted for 10 h.

Mechanical ventilation was resumed and after skin closure, the patient was decannulated from ECMO intraoperatively. The patient was extubated after 24 h postoperatively. Intensified rehabilitation program was done together with intensified pain control measures. The patient was discharged from the ICU after 10 days. Final hospital discharge was after another 10 days. The patient was given oral Posaconazole 300 mg/day for 1 month.

## Discussion

Mucormycosis represents a group of infections caused by the filamentous fungi of the order Mucorales of the subphylum Mucoromycotina (formerly called as Zygomycetes). The term zygomycosis is used synonymously with mucormycosis in the literature [5]. The most common agents of mucormycosis are *Rhizopus* spp., *Mucor* spp., and *Lichtheimia* (formerly *Absidia* and *Mycocladus*) spp. Genera of other Mucorales, such as *Rhizomucor, Saksenaea, Cunninghamella*, and *Apophysomyces*, are less common [2]. Mucormycosis, is considered the third most common invasive fungal infection after candidiasis and aspergillosis [2].

The most important conditions predisposing to mucormycosis, include malignant hematological disease, prolonged and severe neutropenia, poorly controlled diabetes mellitus with or without diabetic ketoacidosis, prolonged use of corticosteroids. Antifungal agents with no activity against Zygomycetes, such as voriconazole and caspofungin, have also been implicated in breakthrough zygomycosis. Iron Overload and Chelation Therapy With Desferrioxamine were reported to increase the risk of mucormycosis especially the disseminated form [6].

Infection primarily occurs by inhalation of its spores and inoculation into the respiratory tract [7]. The spores of mucorales show minimal pathogenicity in immunocompetent hosts because the macrophages can kill spores by phagocytosis and oxidative killing mechanisms. However, macrophages in a host with immune deficiency or diabetes can lose their ability to inhibit spore germination and prevent the hyphae and spores from invading the bronchus and lung [8].

Mucormycosis can present as five predominant forms: rhino-cerebral, pulmonary, cutaneous, gastrointestinal, or disseminated [9]. Rhinocerebral and pulmonary are the most common presentations [10]. Pulmonary forms are reported to be more common in patients with diabetes (49%), hematological malignancies (28%), organ transplant and renal failure (11–12%) [11].

Pulmonary mucormycosis usually resembles bacterial pneumonia with acute onset of fever, cough, hemoptysis, and dyspnea. Pulmonary mucormycosis manifest radiologically as consolidation, cavitation, and less commonly as a solitary pulmonary nodule or mycotic pulmonary artery aneurysms [5]. Endobronchial mucormycosis is very rare and has been sporadically reported in previously published case reports. Diabetes mellitus, steroid therapy and renal insufficiency has been reported as major risk factors. Involvement of the primary bronchus was the most frequently involved location [2], [4], [7], [8], [9], [11]. Bronchoscopically, they mimic a malignant lesion findings a with a gray-white mucoid material and surrounding mucosal edema and necrosis [12]. In many cases, it is postulated that a submucosal, invasive fungal infection causes a submucosal abscess, which presents as an endobronchial mass[13]. The recovery of Zygomycetes can be difficult in culture. Definite diagnosis is always achieved with biopsy and histopathological examination, which reveals a tissue invasion by aseptate broad right-angled branching hyphae with a tendency to invade blood vessels [14].

In our case, our first impression after bronchoscopy was that we are dealing with a case of malignant endobronchial obstruction. The most common cause of malignant endobronchial obstruction is direct extension from an adjacent tumor(most commonly bronchogenic carcinoma, followed by esophageal and thyroid carcinoma). Primary tumors of the airway are relatively uncommon. The majority of primary tracheal tumors are squamous cell carcinoma or adenoid cystic carcinoma. Carcinoid tumors, however, account for the majority of primary airway tumors distal to the carina. Distant tumors may also metastasize to the airway, with the most common causes including renal cell, breast, and thyroid carcinoma [15].

In our case, cryotherapy was chosen as a diagnostic and therapeutic modality. The use of a cryo-probe enhanced removal of large amounts of tissue for biopsy as well as recanalization of the obstructed lumen. Cryoprobe-based therapy is based on the Joule–Thomson physical principle whereby a liquefied gas under pressure that exits through a small orifice undergoes rapid conversion and expansion to the gaseous form. This liquid–gas conversion is accompanied by a dramatic temperature drop that is captured in the cryoprobe tip. Tissue damage occurs when the cryoprobe is brought into contact with the target tissue[16]. In other words, Cryotherapy is the use of extreme cold to destroy tissue using rapid freeze–thaw cycles. Cryotherapy is now being used in several clinical settings, including the treatment of benign and malignant central airway obstruction, transbronchial biopsy, endobronchial biopsy, foreign body removal, and the treatment of low-grade airway malignancy [17].

Histopathological examination, which reveals a tissue invasion by aseptate broad right-angled branching hyphae with a tendency to invade blood vessels is considered the gold standard in diagnosis [14]. This explains what we encountered in the current case with a negative tissue culture and a characteristic histopathology positive for mucormycosis.

Due to poor penetration of antifungals at the site of infection, current guidelines recommend a combined medical and surgical approach to management [18]. Amphotericin B(5–10 mg/kg) is the first line agent in treatment of mucormycosis. However, its potential toxicity especially with high doses and prolonged duration of treatment required sometimes hinders it use [19]. Isavuconazole is a triazole antifungal drug which has also been recommended as the first line agent for mucormycosis with similar efficacy to amphotericin B and similar results making it a good alternative in amphotericin drug toxicity [19]. Oral posaconazole is also recommended as salvage therapy [20].

Surgical treatment in the form of wedge resection, lobectomy, and pneumonectomy, in combination with antifungal therapy, has been associated with lower mortality rates and better outcomes [2], [7]. Surgical resection should be done as soon as feasible to prevent dissemination and erosion into the vessels, resulting in potentially fatal massive hemoptysis [2].

## Funding

No funding was obtained.

## Ethical approval

Administrative authorization and ethical approval were obtained from the ethical committee of the institutional review board of Ministry of Health, Cairo, Egypt (No: 3–2021/19).

## Consent

Written informed consent was obtained from the patient for publication of this case report and any accompanying images. A copy of the written consent is available for review by the Editor-in-Chief of this journal.

## CRediT authorship contribution statement

Conception and design, acquisition of data, diagnosing and follow-up of the patient: HA. SA performed the bronchoscopy and cryobiopsy, AM did the surgery, DA did the histopathological exam. Drafting the article or revising it critically for important intellectual content: HA, MA, NL, HM. All authors read and approved the final manuscript.

## Declaration of Competing Interest

The authors have no conflicts of interest to declare.

## Data Availability

Data sharing is not applicable to this article as no datasets were generated or analyzed during the current study.

## References

[1] Manjunath M., Prajapat D., Sharma R.K., Talwar D. (2018). Refractory bronchovascular pleuropulmonary mucormycosis: case report and difficulties in management. Lung India.

[2] Bajwa A., Hussain S., Youness H., Sawh R., Zhao L., Abdo T. (2022). Endobronchial mucormycosis: a rare clinical entity diagnosed by endobronchial cryobiopsy. Respir Med Case Rep.

[3] Nattusamy L., Kalai U., Hadda V., Mohan A., Guleria R., Madan K. (2017). Bronchoscopic instillation of liposomal amphotericin B in management of nonresponding endobronchial mucormycosis. Lung India.

[4] He R., Hu C., Tang Y., Yang H., Cao L., Niu R. (2018). Report of 12 cases with tracheobronchial mucormycosis and a review. Clin Respir J.

[5] Bigby T.D., Serota M.L., Tierney L.M., Matthay M.A. (1986). Clinical spectrum of pulmonary mucormycosis. Chest.

[6] Petrikkos G., Skiada A., Lortholary O., Roilides E., Walsh T., Kontoyiannis D. (2012). Epidemiology and clinical manifestations of mucormycosis. Clin Infect Dis.

[7] Jaganathan V., Madesh V., Subramanian S., Muthusamy R., Mehta S. (2019). Mucormycosis: an unusual masquerader of an endobronchial tumour. Respirol Case Rep.

[8] Mahishale V., Patil B., Mahishale A., Malur P., Avuthu S., Eti A. (2016). Endobronchial pulmonary mucormycosis diagnosed by fiberoptic bronchoscope: a rare case report. Med J Dr D Y Patil Univ.

[9] Mahajan R., Paul G., Chopra P., Suri P. (2014). Mucormycosis masquerading as an endobronchial tumor. Lung India.

[10] Fermanis G., Matar K., Steele R. (1991). Endobronchial zygomycosis. Aust N Z J Surg.

[11] Maddox L., Long G., Vredenburgh J., Folz R. (2001). Rhizopus presenting as an endobronchial obstruction following bone marrow transplant. Bone Marrow Transpl.

[12] Husari A.W., Jensen W.A., Kirsch C.M. (1992). Pulmonary mucormycosis presenting as an endobronchial lesion. Chest.

[13] Donohue J.F. (1983). Endobronchial mucormycosis. Chest.

[14] Badiee P., Arastefar A., Jafarian H. (2013). Comparison of histopathological analysis, culture and polymerase chain reaction assays to detect mucormycosis in biopsy and blood specimens. Iran J Microbiol.

[15] Ernst A., Feller-Kopman D., Becker H., Mehta A. (2004). Central airway obstruction. Am J Respir Crit Care Med.

[16] Yiu W.K., Basco M.T., Aruny J.E., Cheng S.W., Sumpio B.E. (2007). Cryosurgery: a review. Int J Angiol.

[17] DiBardino D., Lanfranco A., Haas A. (2016). Bronchoscopic cryotherapy clinical applications of the cryoprobe, cryospray, and cryoadhesion. Ann ATS.

[18] Brunet K., Rammaert B. (2020). Mucormycosis treatment: recommendations, latest advances, and perspectives. J Mycol Med.

[19] Cornely O., Alastruey-Izquierdo A., Arenz D., Chen S., Dannaoui E., Hochhegger B. (2019). Global guideline for the diagnosis and management of mucormycosis: an initiative of the European confederation of medical mycology in cooperation with the mycoses study Group education and research consortium. Lancet Infect Dis.

[20] Greenberg R., Mullane K., Van Burik J., Raad I., Abzug M., Anstead G. (2006). Posaconazole as salvage therapy for zygomycosis. Antimicrob Agents Chemother.

